# Protocol for a home-based integrated physical therapy program to reduce falls and improve mobility in people with Parkinson’s disease

**DOI:** 10.1186/1471-2377-12-54

**Published:** 2012-07-16

**Authors:** Meg E Morris, Clarissa Martin, Jennifer L McGinley, Frances E Huxham, Hylton B Menz, Nicholas F Taylor, Mary Danoudis, Jennifer J Watts, Sze-Ee Soh, Andrew H Evans, Malcolm Horne, Peter Kempster

**Affiliations:** 1 Department of Physiotherapy, The University of Melbourne, Carlton, VIC, 3010, Australia; 2 Musculoskeletal Research Centre, Faculty of Health Sciences, La Trobe University, Bundoora, VIC, 3086, Australia; 3 Department of Physiotherapy, Faculty of Health Sciences, La Trobe University, Bundoora, VIC, 3086, Australia; 4 Center for Health Economics, Monash University, Building 75, Clayton, VIC, 3800, Australia; 5 Department of Neurology, Royal Melbourne Hospital, Parkville, VIC, 3052, Australia; 6 Howard Florey Neurosciences Institute, The University of Melbourne, Parkville, VIC, 3010, Australia; 7 Neurosciences Department, Monash Medical Centre, Clayton, VIC, 3168, Australia

**Keywords:** Parkinson’s disease, Accidental falls, Randomized controlled trial, Falls prevention

## Abstract

**Background:**

The high incidence of falls associated with Parkinson’s disease (PD) increases the risk of injuries and immobility and compromises quality of life. Although falls education and strengthening programs have shown some benefit in healthy older people, the ability of physical therapy interventions in home settings to reduce falls and improve mobility in people with Parkinson’s has not been convincingly demonstrated.

**Methods/design:**

180 community living people with PD will be randomly allocated to receive either a home-based integrated rehabilitation program (progressive resistance strength training, movement strategy training and falls education) or a home-based life skills program (control intervention). Both programs comprise one hour of treatment and one hour of structured homework per week over six weeks of home therapy. Blinded assessments occurring before therapy commences, the week after completion of therapy and 12 months following intervention will establish both the immediate and long-term benefits of home-based rehabilitation. The number of falls, number of repeat falls, falls rate and time to first fall will be the primary measures used to quantify outcome. The economic costs associated with injurious falls, and the costs of running the integrated rehabilitation program from a health system perspective will be established. The effects of intervention on motor and global disability and on quality of life will also be examined.

**Discussion:**

This study will provide new evidence on the outcomes and cost effectiveness of home-based movement rehabilitation programs for people living with PD.

**Trial registration:**

The trial is registered on the Australian and New Zealand Clinical Trials Registry (ACTRN12608000390381).

## Background

Most people with idiopathic Parkinson’s disease (PD) live at home or in the community. A major aim of physical therapy is to enable them to remain at home safely for as long as possible [1-3]. Even though it is estimated that more than 7 million people worldwide have PD [4], there have been few investigations of the most effective home-based exercise and rehabilitation programs for those with this debilitating condition. Falls occur often in people with idiopathic PD as the disease progresses [5,6], with fall rates of over 60% per annum reported [7,8]. More than half of these individuals fall more than once every year [8-10]. It appears likely that the cognitive difficulties frequently associated with PD aggravate the risk of falling, particularly by impairing attention [11].

Potential injury is not the only adverse consequence of a fall. Falling may lead to a fear of falling [12,13], which can in turn result in further falls [14,15]. In people with PD, fear of falling has been associated with an increased risk of frequent falls [16]. Falls often lead a person to reduce their activity levels [14,17,18], community ambulation [19] and their participation in outdoor activities [20], leading to loss of fitness and independence.

Falls not only compromise quality of life [21], but can sometimes impact negatively on caregiver quality of life, with reported issues including depression, sustained productivity losses, fear for their spouse, and injuries sustained in trying to prevent their spouse’s fall [22,23]. The costs from health care service utilization following falls are also substantial [24]. A recent study [25] found annual direct costs of medical care in the USA to be $US23,101 per person in people with PD and only $US11,247 in matched controls, noting that falls were substantial contributors to the difference.

There are several approaches to physical therapy that can be delivered within the home. One effective method, known as movement strategy training (MST), teaches the individual to compensate for the disabling movement disorders that occur in PD. Based on studies by Morris and Iansek [2,26,27], these strategies teach people to use attentional strategies to consciously bypass the basal ganglia, instead using the frontal cortex to initiate and execute functional activities. Motor performance is enhanced by the use of structured practice, which breaks down complex movement sequences into segments and focuses attention on each segment before practicing the activity as a whole. Additional components of MST are the mental rehearsal of forthcoming movements, conscious focus on the movement as it occurs, and the use of supplementary visual or auditory cues. While MST can improve the ability to move [28], its ability to prevent falls within the home and community has not yet been confirmed.

Progressive resistance strength training (PRST) is an exercise therapy that can increase the ability of muscles to generate force. Strength is reduced in many people with PD [29,30] most likely because hypokinesia and aging lead to reduced physical activity and disuse. There is preliminary evidence that PRST for people with Parkinson’s can result in increased muscle strength and hypertrophy [31], improved walking ability [30] and enhanced balance [32,33]. Although PRST has been shown to reduce falls in people with musculoskeletal conditions such as osteoporosis [34], it remains to be seen whether PRST can minimize falls and reduce injuries in people with PD who live at home.

Education packages about the causes and prevention of falls have successfully reduced falls incidence in older people without PD, both in acute and sub-acute care [35] and as part of a multi factorial intervention in community dwelling older people [36-38]. Because PD is more prevalent in the aged population, people with this chronic condition are likely to experience many of the same risk factors for falls as their contemporaries, suggesting that such education packages may also be effective. A study of PD by Sadowski et al. [39] found they had little knowledge of the risk factors specific to PD, despite having fallen. Falls prevention programs are arguably cost-effective [40,41], warranting their evaluation in PD.

Preliminary evidence suggests that an integrated program of progressive resistance strength training, movement strategy training, physical activities, and falls education may reduce falls and disability and improve quality of life in those with PD [7,42,43]. Although these interventions have been investigated independently in the PD literature [44-46], the effects of combining them to provide an optimal package remain unclear. A recent report of an integrated program of group strength and balance exercises in 130 community dwelling people with PD did not identify a significant reduction in fall rates [43]; however, there were differences in the incidence rate ratio (IRR) of falls that may have been clinically meaningful (IRR 0.68 during intervention; 0.74 during follow-up). While the intervention utilized exercises previously prescribed for the frail elderly or older recurrent fallers, there was no focus on the specific difficulties encountered in PD, such as turning or gait initiation problems, and no falls education was provided.

Another randomized controlled trial of integrated therapy examined the effect of a 6 weeks home program on falls rate in a sample of 142 community-dwelling individuals with PD who reported falling in the previous year [7]. The intervention of strengthening, flexibility, balance and gait exercises incorporated strategies on falls prevention and for gait initiation and compensation, but no specific falls education program. Despite the addition of some PD-specific activities, there was only a trend to fewer falls and injurious falls in the exercise group, although near falls and repeated falls were significantly decreased in that group.

Most previous trials of exercise therapy in PD have evaluated rehabilitation in the hospital [28] or outpatient clinic setting [47,48]. Therapy provided in the home, such as by Ashburn et al. [7], rather than the clinic may be more appropriate for a relatively frail, generally elderly population as it is convenient, accessible and affordable. Obviating the need for travel may reduce stress on people with PD and the significant others in their lives. Home-based therapy may also allow for treatment of more disabled people, who would have difficulty travelling to a clinic. A less obvious benefit is the specificity of home training. Movement strategy training focuses on improving an individual’s movement difficulties, such as freezing when turning the corner into the bathroom. While practice in a clinical setting may aim to replicate the turn in direction and angle during treatment, only on-site training can be fully specific. There is some evidence that PD reduces the flexibility of motor learning, increasing the need for task-specific practice [49]; thus home-based therapy seems particularly suitable for people with this condition.

The primary aim of the current study is to investigate whether a short (6 weeks) integrated home rehabilitation program (IR) comprising progressive resistance strength training, movement strategy training and falls prevention education reduces falls incidence over 12 months when compared to a home-based control “life skills” group. Secondary aims are to examine the effects of the IR program on disability and quality of life relative to the control group, and to explore the outcome of injurious falls. An economic evaluation from the perspective of the health care system will be carried out alongside the clinical trial.

## Methods/design

### Design

This study will be a prospective controlled trial with random allocation stratified by recruitment source to control for possible bias. Intention to treat analysis will be used and assessors blinded to group allocation. A concurrent economic analysis will evaluate the costs of falls and their management, as well as the costs of implementing the therapy intervention.

### Participants

Community-dwelling people with PD from metropolitan Melbourne will be recruited via hospital-based neurologists and the state Parkinson’s support group if they have a neurologist-confirmed diagnosis of idiopathic PD. Other inclusion criteria are: aged above 21 years; Hoehn and Yahr [50] of < 5, denoting the ability to walk independently with or without an aid; no overt dementia as determined by a Mini-Mental State Examination [51] score of >23/30, age adjusted. Participants must be able to follow simple instructions delivered in English, provide written consent to participation in the study and be medically safe to undertake an exercise program as determined by their neurologist.

### Power calculation and sample size

The primary dependent variable of falls frequency over 12 months was used to determine sample size. Based on previous studies [8,52] and setting power at 80%, alpha at 5% and with a drop-out rate of 20%, a sample size of 90 is required in each group to detect a clinically significant difference of 25% reduction in falls frequency in the intervention group. This sample size will also be sufficient to identify between-group differences in the secondary outcomes of the Movement Disorders Society – Unified Parkinson’s Disease Rating Scale (MDS-UPDRS) [53] and Parkinson’s Disease Questionnaire-39 items (PDQ-39) [54,55].

### Randomization and blinding

One hundred and eighty people with Parkinson’s will be randomly allocated to either the intervention group (integrated rehabilitation program (IR)) or the control group who will participate in life skills information sessions (LS). Randomization will be stratified according to each referral source in permuted blocks of 6, 8 and 10 by an independent private trials coordination organization. Those involved in recruitment and assessment will be blind to group allocation.

### Interventions

The IR program and LS sessions will be delivered in participants’ homes by therapists trained in the appropriate intervention at a standardized training session conducted by the study team. Both IR and LS programs will run for six weeks. Interventions will comprise one 60 minutes session per week delivered by a therapist, plus a 60 minutes “homework” session performed by the participant following pre-printed but individualized worksheets. Physiotherapists will deliver the IR intervention and allied health professionals including occupational therapists and speech pathologists will run the LS sessions. Both the IR program and the LS sessions will occur in addition to participants’ usual healthcare.

#### Integrated rehabilitation program

The IR participants will undertake a structured but individually tailored program of (i) progressive resistance strength training (PRST), (ii) movement strategy training (MST) and (iii) falls prevention education. This protocol integrates elements from interventions previously detailed by Morris et al. [27]. Written advice to therapists will assist them to schedule the time allocated between the various activities. Strength training exercises will be prioritized over other activities if a participant is unable to complete all three components within the allotted time for each session (60 minutes). The “homework” program will repeat activities from the therapist’s session for that week, modified to participant need and any safety issues. Pre-printed forms will be used to report the activities undertaken by each individual at each session and the amount of perceived effort used. Participants will record homework performance on similar forms. These data will enable later evaluation of adherence to and compliance with the interventions. Participants will be asked at each session whether they experienced any negative side effects of treatment such as muscle soreness or joint stiffness from the previous session, whether it required any health service attendance and the results recorded.

Participants will be given an intervention pack containing a booklet with the exercises pictured and described, a modified Rate of Perceived Exertion scale (mRPE) [56], an exercise log book (used to monitor compliance with the homework program), a sheet of frequently asked questions about strength training and a booklet on falls prevention [57]. They will also receive a standard help sheet from Parkinson’s Victoria listing resources for participant or caregiver support.

#### (a) Progressive resistance strength training

Strength training will focus on the major muscle groups integral to functional gait, balance and transfers, such as quadriceps, glutei, hip abductor and trunk muscles. The five exercises incorporated are: sit to stand; step-ups; heel raises; standing hip abduction; and trunk extension and rotation. The training protocol will follow guidelines published by the American College of Sports Medicine to ensure optimal training stimulus [56]. Therapists will train participants how to perform the exercises safely, focusing on correct form. They will also assist participants to understand the level of perceived exertion required, i.e. hard – very hard (5 – 7 on the Modified Rating of Perceived Exertion [56]. Exercise resistance may be progressed by altering starting position or via a weighted vest or resistance bands. Participants will complete 2 sets of 8 to 12 repetitions of each exercise, with 2 minutes rest between each set, aiming for a minimum of three different strengthening exercises each session.

#### (b) Movement strategy training

MST will be based upon the principles previously described in detail by Morris [2,26,27] emphasizing evidence-based training techniques for people with Parkinson’s. These include the ability of participants to initiate and execute everyday functional activities, facilitated as appropriate by the use of cues (visual, auditory, cognitive or proprioceptive) and attentional strategies, such as conscious attention strategies, such as conscious attention. This training approach uses practice of everyday activities such as standing up, moving from chair to chair, standing and reaching, walking, walking while carrying objects or turning and bed mobility. Activities will be selected and progressed according to each participant’s individual ability, needs and home environment. For more detail on possible progression, see Morris et al. (2011) [27].

#### (c) Falls education

Education about falls prevention will be based upon the content of the booklet “Don’t Fall for It! Falls Can Be Prevented” [57]. The Commonwealth of Australia has adopted and published this guide for the prevention of falls in older people. It comprises advice on various aspects of falls and safety, such as an overview of risk factors, keeping mobile, medication, vision, feet and footwear, and safety in the home environment. Each IR session will reflect the booklet content, with particular reference as to how that topic may be relevant to the individual participant.

### Life skills training

The control group’s LS sessions will be of equivalent duration to the IR program, with the same therapist-participant contact time and a weekly self-directed but structured “homework” session. The program will consist of a series of guided education and discussion sessions based on topics of interest. These will be selected by each participant from a pre-defined syllabus to optimize retention and interest. Topics include information on: relaxation; energy conservation; fatigue management; voice and communication; swallowing and diet; travel advice; and memory skills. No session content will relate to physical activity, exercise, walking, or falls risk education. Participants in the LS group will also be given a general intervention pack containing the standard help sheet from Parkinson Victoria, Australia. Ethics required us to provide a generic falls information sheet also.

### Assessments

All assessments will be performed in the participant’s home by a trained physiotherapist blind to group allocation. Participants will be reminded not to disclose their treatment or group allocation to the assessor. Assessments will occur in the week prior to therapy commencing (T1), in the week after six weeks’ therapy (T2) and 12 months after therapy completion (T3). Difference between T1 – T2 assessments will allow evaluation of the immediate effect of therapy, while T2 to T3 difference will determine whether the IR program has a longer term effect. Each assessment will include the MDS-UPDRS [53], PDQ39 [54,55] and the EuroQol EQ-5D^TM^[58]. Medications and their dosage will be recorded on each test occasion.

### Outcome measures

#### Primary

The primary outcome measure will be falls, defined as “an unexpected event in which the participants comes to rest on the ground, floor or lower level” [59]. Falls will be monitored during the 6 weeks intervention and for 12 months thereafter, consistent with the recommendations of the ProFaNE consensus group . Participants will mark all falls on the day they occur on a falls calendar, [60]or mark a month without falls by ticking a box marked “I had no falls”. Pre-paid envelopes will be provided for the return of each month’s calendar sheet. Injurious falls, defined as a fall requiring medical attention or any healthcare utilization, will be marked with a yellow sticker. Telephone calls will be used to remind participants who have not returned their monthly sheet. Calls will also investigate each injurious fall using a set questionnaire that examines the health and economic sequelae of the fall based on data from Medicare Australia, self-reported health care utilization and out-of-pocket expenditure.

#### Secondary

Secondary outcome measures include changes in motor disability, global disability and quality of life. Changes in motor disability and global disability will be compared from pre- to post-therapy (T1 to T2) and from T2 to 12 months after therapy ceases (T3), using respectively the motor section III of the MDS-UPDRS [53,61] and the full MDS-UPDRS. The MDS-UPDRS is the current version of the original UPDRS [62], augmented and revised in line with the findings of its review taskforce (MDS, 2003), and represents the gold standard in PD assessment. Quality of life will be evaluated on the same occasions, using the disease-specific PDQ-39 [54,55] which has demonstrated validity, reliability and sensitivity. The widely used generic EQ-5D [58] will allow comparison of quality of life with non-PD populations [63].

### Economic evaluation

To enable calculation of the costs of the intervention relative to the number of falls prevented, the direct costs of implementing the therapy programs including the costs of training therapists will be established. The additional information collected after injurious falls will allow calculation of the medical, medical ancillary, diagnostic and hospitalization costs associated with these falls in the 12 months follow-up period. An incremental cost effectiveness ratio (ICER) will be calculated for the primary outcome as the difference in total cost per fall prevented between the IR and LS groups. For the secondary outcomes the ICER will be calculated for cost per injurious fall prevented and cost per quality adjusted life year saved.

### Statistical analysis

#### Primary

Falls, as the primary variable of interest, will be examined in four ways consistent with the recommendations of the ProFaNe group [60]:

i. number of fallers

ii. number of multiple fallers (two or more falls)

iii. falls rate and time to first fall

iv. relative risk (RR) will be calculated to compare number of fallers and number of multiple fallers between the two groups.

The number of falls and the falls rate per person per year in the two groups will then be compared using negative binomial regression models, an approach that takes into account all falls and adjusts for varying duration of follow up. Injurious falls, as defined above, will be analyzed in the same way.

#### Secondary

All variables will be examined using descriptive statistics to ensure they meet the assumptions necessary for parametric analysis. If not, non-parametric equivalent analyses will be used. Repeated measures ANOVA with planned comparisons will examine between group differences in the secondary outcome variables and investigate the effects of the intervention.

### Trial registration and ethics approval

Ethical approval has been granted from the University of Melbourne Human Research and Ethics Committee (0824406). All participants will provide written informed consent. The trial is registered on the Australian and New Zealand Clinical Trials Registry (ACTRN12608000390381).

## Discussion

This randomized controlled trial will establish the outcomes and cost effectiveness of a short integrated rehabilitation program of home-based therapy for people with PD that integrates PRST**,** MST and falls education. Providing therapy in the home may enhance participation in the study and allow people with PD with a range of disabilities and levels of progression to access rehabilitation programs. The results will build on those from our earlier study [27]. This compared the ability of MST with falls education, and PRST with falls education to reduce falls each relative to a control group who received life skills education only. The results from the current project will provide further information on the effectiveness of home-based programs for people living with PD.

## Abbreviations

PD, Parkinson’s disease; H&Y, Hoehn and Yahr; MDS-UPDRS, Movement Disorders Society-Unified Parkinson’s Disease Rating Scale; PDQ-39, Parkinsons’ Disease Questionnaire – 39 items; EQ-5D, EuroQol-5D; mRPE, Modified rate of perceived exertion; IR, Integrated rehabilitation; LS, Life skills; MST, Movement strategy training; PRST, Progressive resistance strength training; ICER, Incremental cost effectiveness ratio.

## Competing interests

The authors declare that they have no competing interests.

## Authors’ contributions

MM, JMcG, FH, HM, JW, NT, AE, MH, PK conceived the idea for the study and participated in the design of the study. CM and MD acted as project managers and participated in subject recruitment and drafting of the manuscript. MM, JMcG, FH, S-E S and HB drafted the manuscript for submission and are involved in knowledge translation to clinicians. All the authors have read and approved the manuscript.

## Pre-publication history

The pre-publication history for this paper can be accessed here:

http://www.biomedcentral.com/1471-2377/12/54/prepub
